# Chromatin remodeling factor, INO80, inhibits *PMAIP1* in renal tubular cells via exchange of histone variant H2A.Z. for H2A

**DOI:** 10.1038/s41598-023-40540-8

**Published:** 2023-08-14

**Authors:** Rika Miura, Imari Mimura, Hanako Saigusa, Tomotaka Yamazaki, Fumiaki Tanemoto, Yu Kurata, Dai Sato, Tetsuhiro Tanaka, Masaomi Nangaku

**Affiliations:** 1 Division of Nephrology and Endocrinology, The University of Tokyo School of Medicine, 7-3-1 Hongo, Bunkyo-ku, Tokyo, 113-8655 Japan; 2 Department of Nephrology, Rheumatology and Endocrinology, Tohoku University Graduate School of Medicine, Sendai, 980-8574 Japan

**Keywords:** Molecular biology, Nephrology

## Abstract

Epigenetic modifications such as DNA methylation, histone modifications, and chromatin structures in the kidney contribute towards the progression of chronic kidney disease (CKD). In this study, the role of chromatin remodeling factor inositol requiring 80 (INO80) was investigated. Although INO80 regulates transcription by altering the chromatin structure at the nucleosome level, its role in the kidney remains unknown. We demonstrated that the expression of *INO80* in impaired kidneys decreased in rats with unilateral urethral obstruction. We investigated INO80 expression in a proximal tubular cell line and observed that its expression decreased under hypoxic condition. Additionally, INO80 knockdown promoted apoptosis, suggesting that INO80 plays a role in inhibiting tubular cell apoptosis. We identified downstream target genes of INO80 via genome-wide analysis using RNA-sequences and found that the expression of apoptosis-related genes, such as *TP53* and *E2F1*, and pro-apoptotic genes, such as *PMAIP1,* increased upon INO80 knockdown. ChIP-qPCR of the loci of *PMAIP1* showed that the amount of H2A.Z. increased instead of decreasing the amount of H2A when *INO80* was knocked down. These results indicated that INO80 plays a role in the exchange of H2A.Z. for H2A in the promoter region of *PMAIP1* in tubular cells to inhibit apoptosis during CKD progression.

## Introduction

Inositol requiring 80 (INO80) is an ATP-dependent chromatin-remodeling factor that plays a role in transcription, DNA repair, and replication. In the eukaryotic nucleus, genomic DNA, the carrier of genetic information, wraps around histone octamers consisting of four core histones (H2A, H2B, H3, and H4) to form nucleosomes. The N- and C-terminal regions of histones are called histone tails and have been reported to undergo various post-translational modifications such as acetylation, methylation, phosphorylation, and mono-ubiquitination^1^. These modifications are thought to be involved in the epigenetic regulation of gene expression by altering chromatin structure. The highly aggregated chromatin structure inhibits the binding of regulatory factors such as basic transcription and replication factors; thus, genomic function is inactivated by chromatin structure. Therefore, genome function is controlled by a chromatin structural transformation, known as chromatin remodeling, which slides and removes nucleosomes. An important function of the chromatin-remodeling complex is to bind histones and work with histone chaperones to remove all or part of the core histones from the nucleosome. Thus, chromatin remodeling factors can positively and negatively control DNA reactions such as transcription, replication, repair, and recombination.

The chromatin-remodeling complex is a large complex composed of multiple subunits. It is classified into four families SWI/SNF (switch/sucrose-non-fermenting); ISWI (imitation switch); CHD (chromodomain-helicase-DNA binding); and INO80—according to the domain constitution type of the Snf2 family protein contained in the complex^2^. In yeast INO80, the histone variant H2A.Z. is genome-wide and controls its dynamics. INO80 has histone exchange activity and has been shown to replace the nucleosome H2A.Z./H2B with free H2A/H2B dimers^3,4^. The H2A.Z. histone variant is located on particular on nucleosomes located on both sides of the promoter region of a gene transcribed by RNA polymerase II; it is also localized on nucleosomes on both sides of the chromatin boundary elements, centromeres, and DNA replication origins^5,6^. These nucleosomes are characterized by rapid turnover independent of DNA replication. Nucleosome turnover is a dynamic process, thought to contribute to epigenome plasticity by controlling gene expression and eliminating histone modifications^7,8^. Histone H2A.Z. promotes the rapid turnover of these nucleosomes.

INO80 has been recently identified in genome-wide studies as a factor affecting kidney function^9^. A meta-analysis of 45 studies identified six single nucleotide polymorphisms (SNPs) that were associated with chronic kidney disease (CKD) risk factors and progression. The SNP (rs2928148) at the INO80 locus is one of these and has been shown to be strongly associated with the CKD risk factor stratum. We examined the relationship between *INO80* expression and CKD to elucidate its role in patients with CKD.

Tubulointerstitial hypoxia is the final common pathway in CKD that promotes interstitial fibrosis^10,11^. In patients with CKD, various factors contributes to chronic hypoxia in the kidney. In CKD, due to glomerulosclerosis and tubulointerstitial damage, physical blood flow in the peritubular capillaries (PTCs) is reduced^12^. In addition, decreased functional blood flow in the PTCs, associated with an increased renin-angiotensin system, causes ischemia of tubulointerstitial cells and decreases the oxygen supply. Renal anemia, a common complication of CKD, can also reduce kidney oxygen supply^11^. Based on previous microarray results, the expression of *INO80* decreases under hypoxia in renal proximal tubule cells. In the present study, we investigated the relationship between tissue hypoxia and *INO80* expression. Here, we clarified the important functions of INO80 in CKD progression.

## Results

### INO80 decreased under hypoxia in HK-2 cells

**Figure 1:**
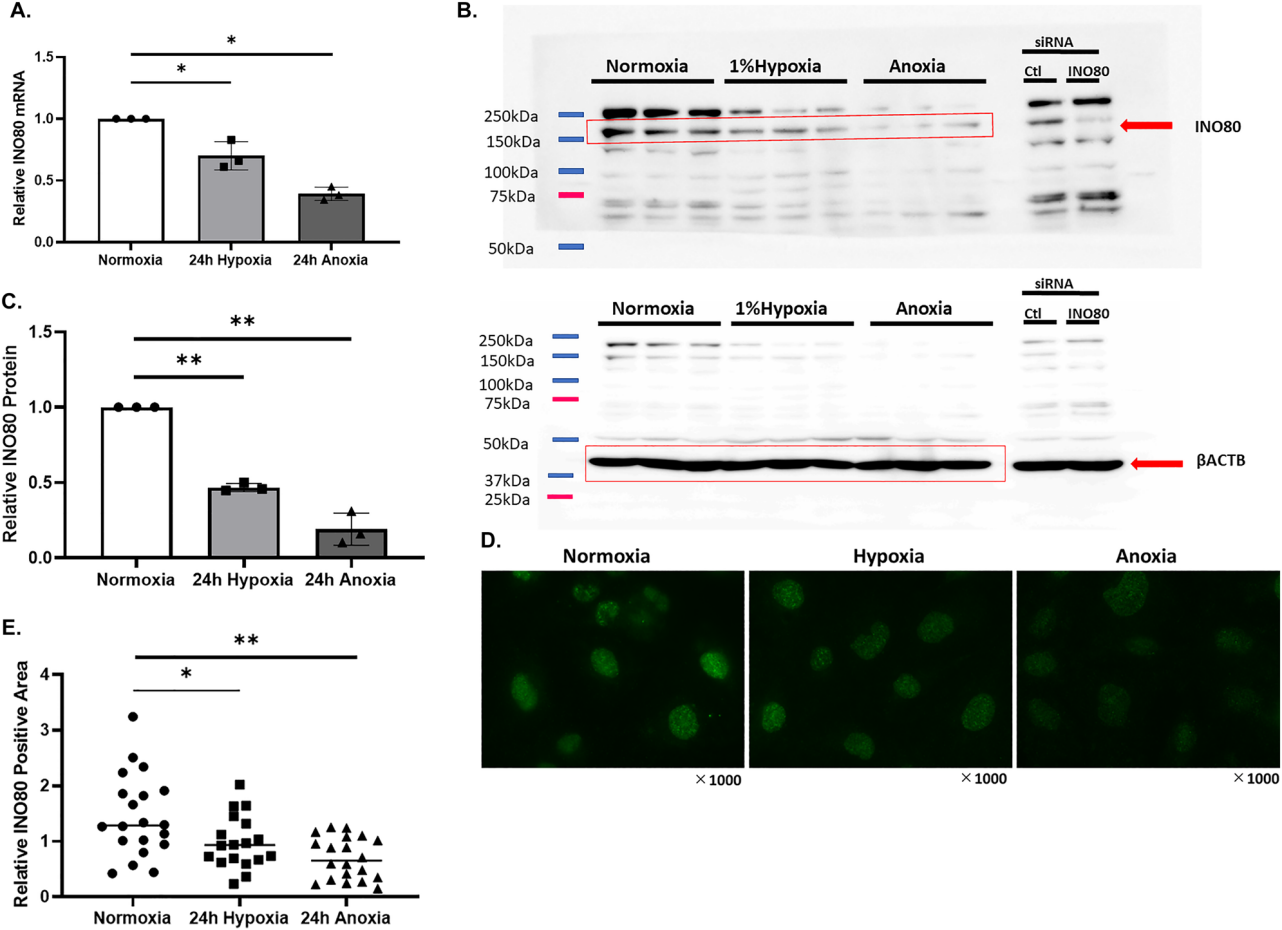
INO80 decreased under hypoxia in HK-2 cells. (**A**) Quantitative PCR analysis of INO80 mRNA expression under normoxia, hypoxia and anoxia for 24 h in HK-2 cells. Data are shown as the mean and the standard deviation from triplicates. *p < 0.05. (**B**) Western blotting of INO80 protein and β-actin extracted from HK-2 cells under normoxia, 1% hypoxia and anoxia for 24 h. The cropped blots were used in the figure and different molecular weight were separated by lines. Full-length blots are presented in Supplementary Figure S2A,B. (**C**) Quantitative analysis of western blot of INO80 under normoxia, 24 h hypoxia and 24 h anoxia. The numbers in the data are the INO80 bands normalized with β-actin. The data uses HK-2 cell samples collected on three different days. **p < 0.01. (**D**) Immunocytochemistry image of INO80 in HK-2 cells under normoxia, 24 h hypoxia, and 24 h anoxia. (**E**) In the immunocytochemistry image of INO80, the fluorescence signal intensity in each nucleus was measured by ImageJ software (N = 20 for each group). *p < 0.05, **p < 0.01.

We evaluated the expression of *INO80* in the immortalized proximal tubular cell line HK-2. When HK-2 cells were cultured at a concentration of 1% hypoxia and 0.1% anoxia for 24 h, *VEGF* (vascular endothelial growth factor) is one of the well-known downstream target genes of HIF-1, and the expression of *VEGF* increases under hypoxia. We confirmed the expression levels of *VEGF* to determine whether hypoxic stimulation was effective (Supplementary Fig. S1). The expression levels of *VEGF* were significantly up-regulated by 1.62-fold and 9.95-fold under 24 h of hypoxia and anoxia, respectively. We examined the mRNA levels of *INO80* under hypoxic conditions and compared them with those under normoxic condition. The expression level of *INO80* decreased 0.7 and 0.39-fold under 24 h hypoxia and anoxia, respectively, compared to that under normoxia (Fig. 1A). *INO80* mRNA levels decreased when cells were cultured under anoxic conditions, rather than under 1% hypoxia. Next, we performed western blotting and immunocytochemistry on HK-2 cells to evaluate INO80 protein expression. Western blotting showed that the INO80 protein level decreased when cultured under hypoxia owing to band quantification using Image-J software; 24 h hypoxia averaged 0.47 and 24 h anoxia averaged 0.19, compared to normoxia (averaged 1.0) (Fig. 1B,C). We showed full membranes of western blotting for shorter exposure times including 8 s, 1 min and 4 min for INO80 and 8 s and 1 min for β-ACTB (Supplementary Fig. S2). For immunocytochemistry, the nuclei of HK-2 cells were stained with the INO80 antibody (Fig. 1D). When HK-2 cells were cultured under hypoxic conditions, fluorescence significantly decreased depending on the oxygen concentration (*p* < 0.05). The analysis of the results revealed that the expression level of INO80 decreased depending on the oxygen concentration. The 24 h hypoxia was 0.67-fold lower than normoxia, and 24 h anoxia was 0.48-fold lower than normoxia. (Fig. 1E). These results indicated that when HK-2 cells were cultured under hypoxia, expression of INO80 decreased at both the mRNA and protein levels.

### INO80 expression decreased in impaired kidneys

**Figure 2:**
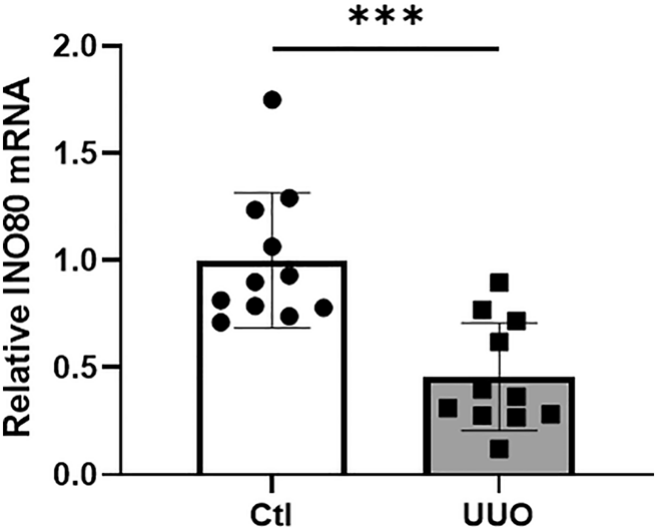
INO80 decreased in in vivo model. Quantitative PCR analysis of *INO80* mRNA expression in UUO model. ***p < 0.001.

We evaluated the expression level of *INO80 *in vivo in rats with impaired kidneys. Rats in the impaired kidney model showed unilateral urethral obstruction (UUO). Ureteral obstruction results in marked renal hemodynamic and metabolic changes, followed by tubular injury and cell death by apoptosis or necrosis with interstitial macrophage infiltration. The UUO model exhibits progressive renal fibrosis. Proliferation of interstitial fibroblasts with myofibroblast transformation leads to excess deposition of the extracellular matrix and renal fibrosis^13^. The mRNA level of INO80 extracted from the whole kidney in the UUO model rat group (n = 6) was significantly reduced compared to the contralateral kidney group (n = 6) (*p* value < 0.01) (Fig. 2).

Experiments were also performed on 5/6 Nx rats. The 5/6 Nx model is often used for studies of CKD. In 5/6 Nx rats, INO80 mRNA expression tended to decrease compared to that in sham rats; however, the difference was not significant (Supplementary Fig. S3A). The extent of interstitial fibrosis was determined by Masson’s trichrome (MT) staining. MT staining showed that interstitial cells and collagen fibers increased in UUO mice. MT staining increased the number of interstitial cells and collagen fibers in 5/6 Nx rats (Supplementary Fig. S3B). Immunohistochemistry revealed that INO80 was mainly expressed in the nuclei of the tubules (Supplementary Fig. S3C). The tubule, especially its nucleus, was strongly stained, similar to UUO rats. Nuclear staining was more intense in sham rats than in the 5/6 Nx rats. We also performed immunohistochemistry on commercially available human kidney samples using an INO80 antibody (Supplementary Fig. S4). Details of the patients’ backgrounds are shown in Supplementary Table S1. Normal kidney samples had more INO80 staining in the cytoplasm compared with CKD patient samples; however, statistical analysis was not possible because only two patients were available. The expression pattern of INO80 in immunohistochemistry was consistent with that of normal kidney samples, with INO80 expression in the tubules being higher than in CKD samples.

### INO80 inhibits apoptosis in HK-2 cells

**Figure 3:**
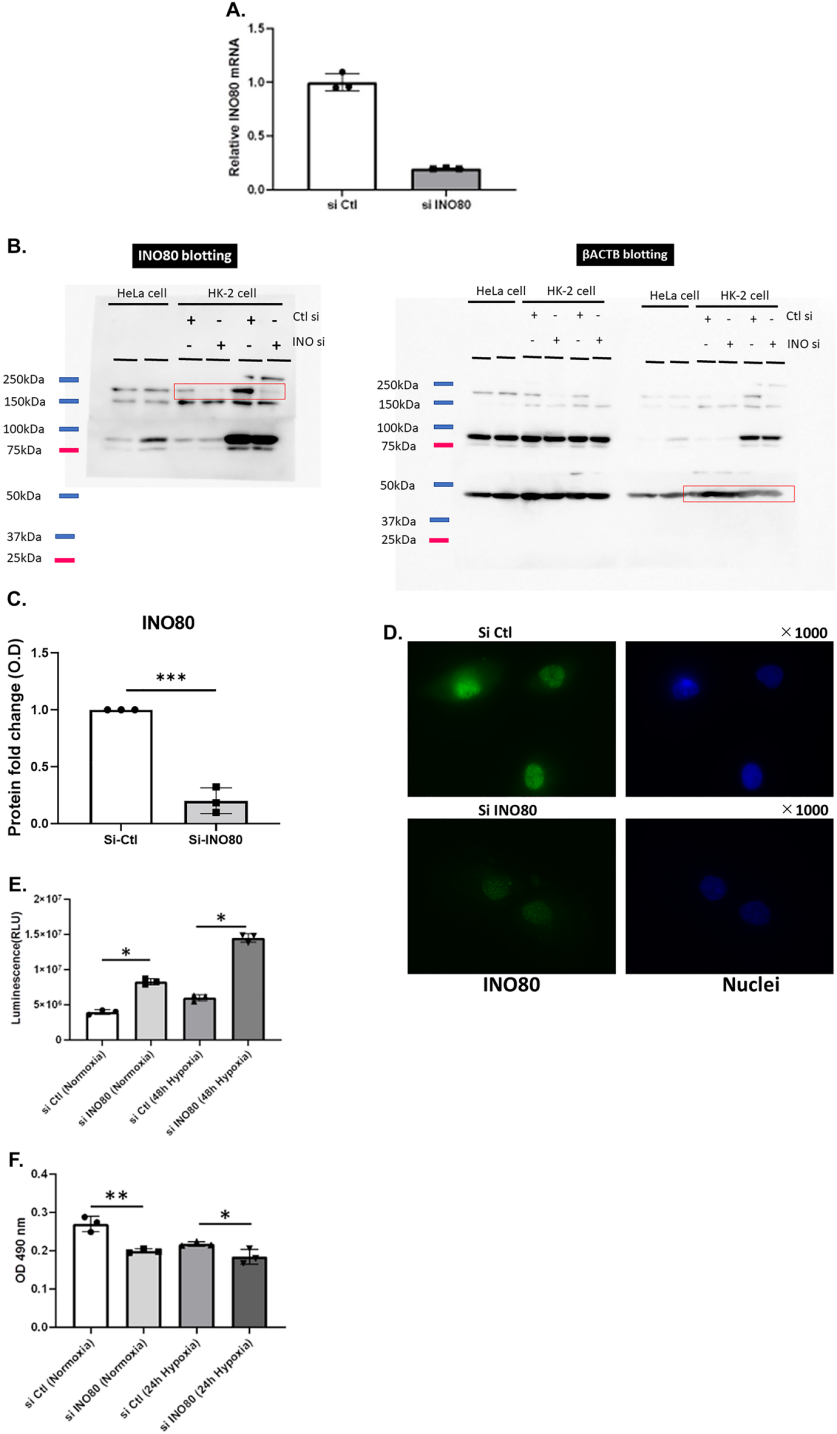
Functional assay of INO80 in HK-2 cells. (**A**) Quantitative PCR analysis of *INO80* mRNA expression in transfected HK-2 cells. (**B**) Full membrane of western blotting of INO80 when INO80 was knocked down using siRNA. The membrane was once cut when INO80 antibody and β-actin antibody were exposed. After the exposure of second antibodies, the separate membranes were lined up next to each other. Ctl means Control siRNA. (**C**) Immunocytochemistry image of INO80 in HK-2 cells when INO80 was knocked down using siRNA. The values in the western-blotting data show the INO80 bands normalized with β-actin. ***p < 0.001. (**D**) The apoptotic rate of HK-2 cells was determined by caspase activation using Caspase 3/7 Glo assay after transfected with negative control or INO80 siRNA for 48 h normoxia or hypoxia. *p < 0.05. (**E**) HK-2 cells viability measured by MTS assay after transfected with negative control or INO80 siRNA for 24 h normoxia or hypoxia. *p < 0.05, **p < 0.01.

To clarify the function of INO80 in tubule cells, we knocked down *INO80* expression in HK-2 cells via siRNA transfection. The knockdown efficiency of INO80 was assessed using quantitative PCR. The transfection efficiency of INO80 was 80.2% in HK-2 cells (Fig. 3A). We also confirmed that INO80 protein expression was reduced by siRNA as shown by western-blotting (Fig. 3B,C) and immunocytochemistry (Fig. 3D). We showed full membranes of western blotting for shorter exposure times including 15 s, 1 min and 4 min for INO80 and 15 s and 1 min for β-ACTB (Supplementary Fig. S5). We performed two different types of assays using HK2 cells. Caspase activity using the Caspase-Glo^®^ 3/7 Assay kit showed that in both normoxia and hypoxia, caspase activity significantly increased in HK-2 cells knocked down with INO80 compared to negative controls [in normoxia, 3,940,380 ± 336,432.1 (siCtl) vs. 8,280,217 ± 415,645.9 (siINO80), *p* < 0.05; in hypoxia, 5,988,033 ± 462,039.8 (siCtl) vs. 14,489,827 ± 605,548.6 (siINO80), *p* < 0.05] (Fig. 3E). The MTS assay detected cell viability when INO80 was knocked down. In both normoxia and hypoxia, viability significantly decreased when INO80 was knocked down [in normoxia, 0.270 ± 0.0198 (siCtl) vs. 0.199 ± 0.0059 (siINO80), *p* < 0.01; in hypoxia, 0.218 ± 0.0059 (siCtl) vs. 0.184 ± 0.019 (siINO80), *p* < 0.05] (Fig. 3F). These results suggested that INO80 plays a role of inhibiting tubule cell apoptosis.

### Genome-wide RNA-seq using HK2 cells knocked down with INO80 identified 32 down-regulated genes and three up-regulated genes

**Figure 4:**
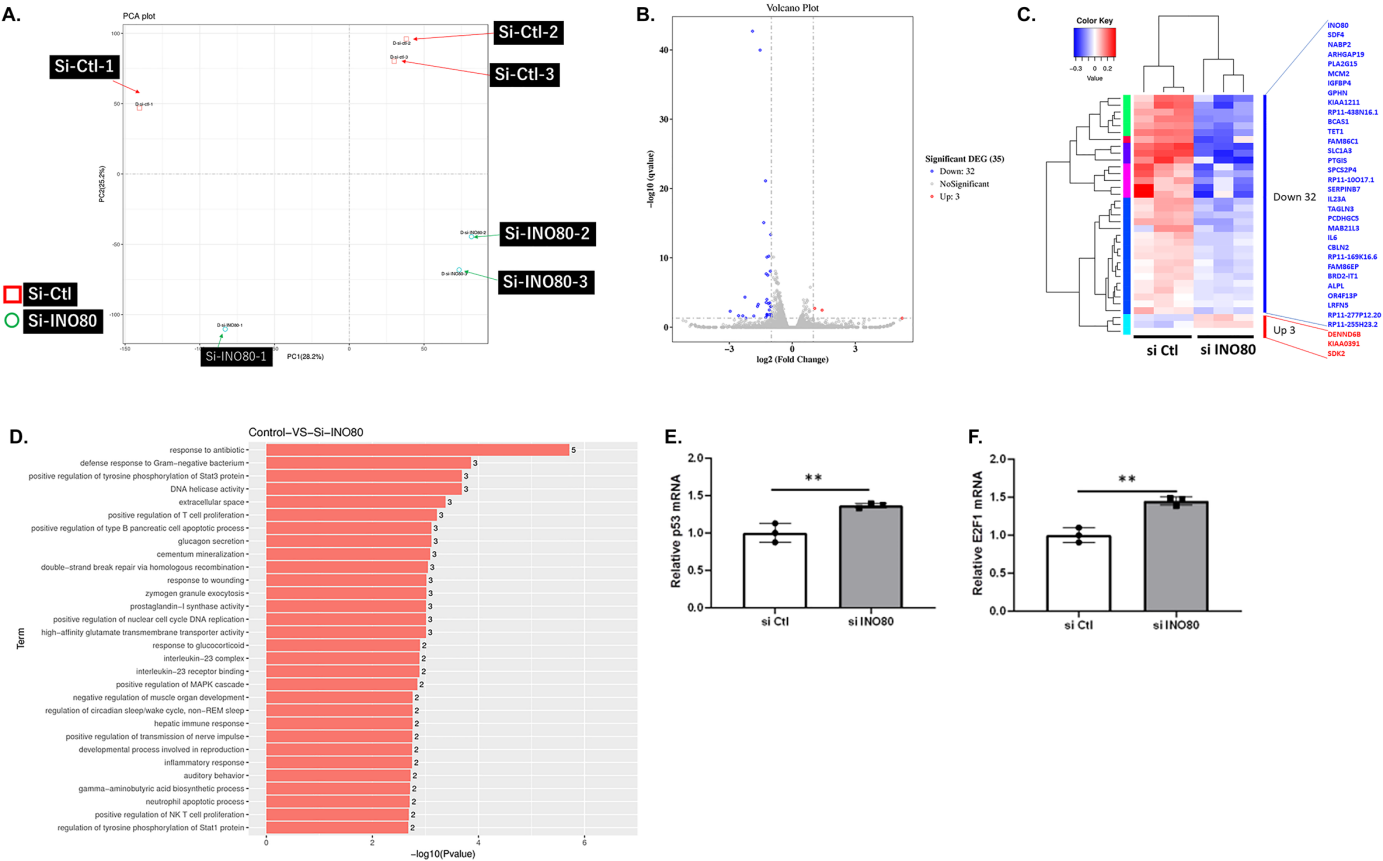
Results of RNA-seq of INO knockdown by siRNA. (**A**) Principal Component Analysis chart. Control siRNA (N = 3) and siRNA of INO80 (N = 3) were analyzed as RNA-seq samples. (**B**) Differential expression volcano plot of each sample. Blue dots were significant down-regulation by siRNA of INO80. Only three red plots were upregulated by INO80 siRNA. X-axis; log 2 folds change of gene expression. Y-axis; statistical significance of differential expression in log 10. (**C**) Cluster analysis of differentially expressed genes log_10_(FPKM + 1) values are used for clustering. Genes of high expressed are in red, and those with low expression are shown in blue. Thirty-five genes were significantly up-regulated (three genes) and down-regulated (thirty-two genes) by INO80 siRNA. The names of these genes have listed. Their expression levels were shown as heatmaps. (**D**) GO enrichment *p* value histogram. X axis: log_10_ (*p* value) of each term. Y axis represents significantly enriched GO terms. The highest *p* value was listed in the top of the graphs in Gene Ontology. (**E**) Quantitative PCR analysis of *TP53* mRNA expression in INO80 knockdown cells. (**F**) Quantitative PCR analysis of *E2F1* mRNA expression in INO80 knockdown cells.

To identify the downstream target genes of INO80, we performed RNA-seq using siRNA of INO80 (N = 3). A principal component analysis (PCA) plot was shown in Fig. 4A. The volcano plot shows the degree of variation in the 35 genes (Fig. 4B). The fluctuation increases with distance from the center to the left and right (horizontal axis). Gene at the top of the figure show significant differences (vertical axis). As shown in the heatmap in Fig. 4C, we identified 32 down-regulated genes (blue) and three up-regulated genes (red; DENND6B, KIAA0391, SDK2) according to the criteria we set. These genes were suspected to be INO80 downstream target candidates. The expression profiles of these genes in the heatmap are shown in Table 1. As a result of gene ontology (GO) (Fig. 4D), apoptosis-related genes such as *TP53* (tumor protein p53), *E2F1* (E2F transcription factor 1), and *PMAIP1* (phobol-12-myristate-13-acetate-induced protein 1, also known as *NOXA*) were significantly associated with INO80 functions. To validate the GO results, we examined the mRNA levels of apoptosis-related genes. As shown in Fig. 4E, the expression of *TP53* mRNA was significantly increased by *INO80* knockdown. It has been reported that INO80 suppresses the expression of the downstream gene of the transcription factor *E2F* in human umbilical venous endothelial cell (HUVEC)^14^, and E2F is involved in the apoptotic pathway, which is different from the p53 pathway. We also demonstrated that the expression of *E2F1* mRNA significantly increased by *INO80* knockdown (Fig. 4F).

**Table 1:** The expression profiles of downstream target gene candidates of INO80 in the heatmap

GeneID	D-si-ctl-1.FPKM	D-si-ctl-2.FPKM	D-si-ctl-3.FPKM	D-si-INO80-1.FPKM	D-si-INO80-2.FPKM	D-si-INO80-3.FPKM	Regulation	Chr	Start	End	Strand	GeneSymbol	Description
ENSG00000128908	6.43	7.07	7.75	1.91	1.75	2.06	Down	15	41,271,078	41,408,552	−	INO80	INO80 complex subunit [Source:HGNC Symbol%3BAcc:26956]
ENSG00000078808	53.78	57.06	57.6	16.8	20.84	20.59	Down	1	1,152,288	1,167,411	−	SDF4	stromal cell derived factor 4 [Source:HGNC Symbol%3BAcc:24188]
ENSG00000139579	14.12	14.65	14.58	5.52	6.62	5.76	Down	12	56,615,799	56,623,638	+	NABP2	nucleic acid binding protein 2 [Source:HGNC Symbol%3BAcc:28412]
ENSG00000213390	2.96	3.16	3.27	1	1.47	1.19	Down	10	98,978,996	99,052,413	−	ARHGAP19	Rho GTPase activating protein 19 [Source:HGNC Symbol%3BAcc:23724]
ENSG00000103066	13.67	16.3	16.54	7.61	7.84	7.35	Down	16	68,279,207	68,294,961	+	PLA2G15	phospholipase A2%2C group XV [Source:HGNC Symbol%3BAcc:17163]
ENSG00000073111	41.83	57.34	54.45	18.92	26.31	25.45	Down	3	1.27E + 08	1.27E + 08	+	MCM2	minichromosome maintenance complex component 2 [Source:HGNC Symbol%3BAcc:6944]
ENSG00000141753	4.03	4.05	4.73	1.79	1.96	1.79	Down	17	38,599,702	38,613,983	+	IGFBP4	insulin-like growth factor binding protein 4 [Source:HGNC Symbol%3BAcc:5473]
ENSG00000171723	2.13	2.62	2.7	1.05	1.29	1.27	Down	14	66,974,125	67,648,520	+	GPHN	gephyrin [Source:HGNC Symbol%3BAcc:15465]
ENSG00000109265	0.94	1.14	1.08	0.46	0.47	0.4	Down	4	57,036,361	57,194,791	+	KIAA1211	KIAA1211 [Source:HGNC Symbol%3BAcc:29219]
ENSG00000249550	2.01	1.99	1.87	0.77	1.08	0.76	Down	12	1.14E + 08	1.14E + 08	−	RP11-438N16.1	–
ENSG00000064787	0.55	0.31	0.34	0.14	0.07	0.03	Down	20	52,553,316	52,687,304	−	BCAS1	breast carcinoma amplified sequence 1 [Source:HGNC Symbol%3BAcc:974]
ENSG00000138336	0.41	0.59	0.54	0.16	0.22	0.29	Down	10	70,320,413	70,454,239	+	TET1	tet methylcytosine dioxygenase 1 [Source:HGNC Symbol%3BAcc:29484]
ENSG00000158483	0.98	1.37	1.35	0.33	0.65	0.58	Down	11	71,498,556	71,512,282	+	FAM86C1	family with sequence similarity 86%2C member C1 [Source:HGNC Symbol%3BAcc:25561]
ENSG00000079215	0.6	0.72	0.9	0.33	0.4	0.32	Down	5	36,606,457	36,688,436	+	SLC1A3	solute carrier family 1 (glial high affinity glutamate transporter)%2C member 3 [Source:HGNC Symbol%3BAcc:10941]
ENSG00000124212	0.54	0.52	0.56	0.2	0.29	0.25	Down	20	48,120,411	48,184,683	−	PTGIS	prostaglandin I2 (prostacyclin) synthase [Source:HGNC Symbol%3BAcc:9603]
ENSG00000228589	4.53	6.19	4.7	1.18	1.03	2.8	Down	1	28,421,582	28,422,933	−	SPCS2P4	signal peptidase complex subunit 2 homolog (S. cerevisiae) pseudogene 4 [Source:HGNC Symbol%3BAcc:45237]
ENSG00000260103	0.33	0.38	0.4	0.07	0.12	0.15	Down	15	74,770,411	74,782,627	−	RP11-10O17.1	–
ENSG00000166396	4.62	3.55	4.19	2.66	1.62	1.85	Down	18	61,420,169	61,472,604	+	SERPINB7	serpin peptidase inhibitor%2C clade B (ovalbumin)%2C member 7 [Source:HGNC Symbol%3BAcc:13902]
ENSG00000110944	9.19	6.9	5.85	4.04	3.72	2.95	Down	12	56,732,663	56,734,193	+	IL23A	interleukin 23%2C alpha subunit p19 [Source:HGNC Symbol%3BAcc:15488]
ENSG00000144834	11.25	8.74	7.38	5.83	3.67	3.29	Down	3	1.12E + 08	1.12E + 08	+	TAGLN3	transgelin 3 [Source:HGNC Symbol%3BAcc:29868]
ENSG00000240764	0.09	0.16	0.07	0	0.03	0.01	Down	5	1.41E + 08	1.41E + 08	+	PCDHGC5	protocadherin gamma subfamily C%2C 5 [Source:HGNC Symbol%3BAcc:8718]
ENSG00000173212	14.03	7.08	7.36	5.64	3.52	3.11	Down	1	1.17E + 08	1.17E + 08	+	MAB21L3	mab-21-like 3 (C. elegans) [Source:HGNC Symbol%3BAcc:26787]
ENSG00000136244	82.3	49.69	45.15	40.35	22.63	19.91	Down	7	22,765,503	22,771,621	+	IL6	interleukin 6 (interferon%2C beta 2) [Source:HGNC Symbol%3BAcc:6018]
ENSG00000141668	0.28	0.46	0.47	0.16	0.12	0.24	Down	18	70,203,915	70,305,756	−	CBLN2	cerebellin 2 precursor [Source:HGNC Symbol%3BAcc:1544]
ENSG00000224321	8.97	10.05	10.31	3.75	7.21	3.52	Down	1	16,119,291	16,119,780	+	RP11-169K16.6	–
ENSG00000251669	0.41	0.59	0.61	0.14	0.21	0.35	Down	4	3,943,487	3,957,146	−	FAM86EP	family with sequence similarity 86%2C member E%2C pseudogene [Source:HGNC Symbol%3BAcc:28017]
ENSG00000223837	0.88	1.6	1.54	0	0.12	0.5	Down	6	32,938,009	32,938,663	+	BRD2-IT1	BRD2 intronic transcript 1 (non-protein coding) [Source:HGNC Symbol%3BAcc:41311]
ENSG00000162551	0.18	0.83	0.83	0.02	0.13	0.21	Down	1	21,835,858	21,904,905	+	ALPL	alkaline phosphatase%2C liver/bone/kidney [Source:HGNC Symbol%3BAcc:438]
ENSG00000214344	0.29	0.36	0.43	0.05	0.18	0.06	Down	15	1.02E + 08	1.02E + 08	+	OR4F13P	olfactory receptor%2C family 4%2C subfamily F%2C member 13 pseudogene [Source:HGNC Symbol%3BAcc:15076]
ENSG00000165379	0.45	0.42	0.28	0.2	0.17	0.19	Down	14	42,076,773	42,373,752	+	LRFN5	leucine rich repeat and fibronectin type III domain containing 5 [Source:HGNC Symbol%3BAcc:20360]
ENSG00000245648	0.4	0.57	0.37	0.17	0.18	0.21	Down	12	10,516,368	10,551,105	+	RP11-277P12.20	–
ENSG00000233836	0.2	0.3	0.2	0.02	0.08	0.04	Down	19	23,945,746	24,016,116	+	RP11-255H23.2	–
ENSG00000205593	0.32	0.28	0.27	0.72	0.49	0.62	Ups	22	50,747,459	50,765,489	−	DENND6B	DENN/MADD domain containing 6B [Source:HGNC Symbol%3BAcc:32690]
ENSG00000258790	0.15	0.09	0.26	0.46	0.45	0.43	Ups	14	35,591,755	35,786,680	+	KIAA0391	Mitochondrial ribonuclease P protein 3 [Source:UniProtKB/TrEMBL%3BAcc:S4R416]
ENSG00000069188	0	0	0	0.02	0.02	0.02	Ups	17	71,330,523	71,640,228	−	SDK2	sidekick cell adhesion molecule 2 [Source:HGNC Symbol%3BAcc:19308]

### INO80 exchanges H2A.Z. for H2A in the promoter region of *PMAIP1*

**Figure 5:**
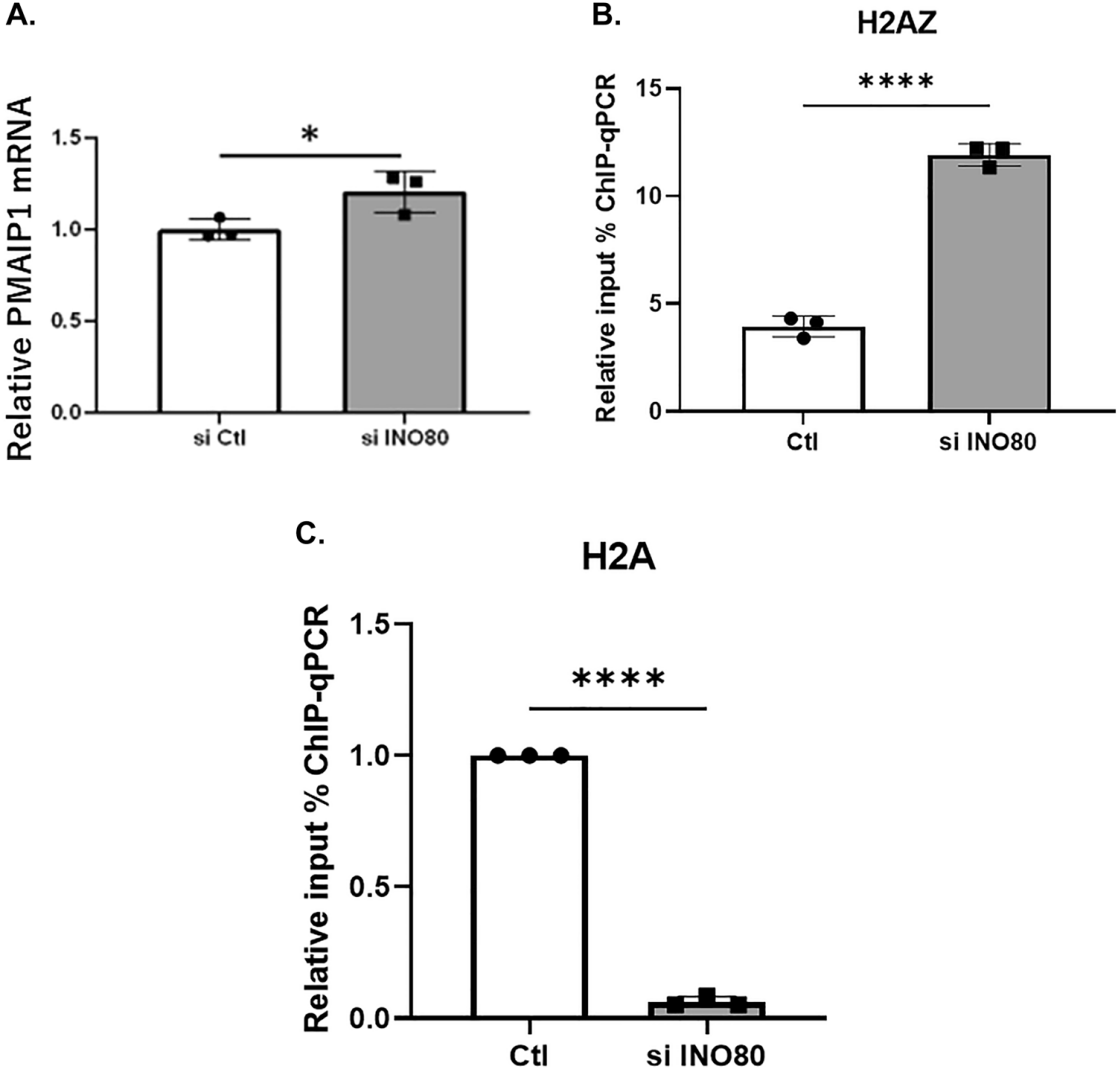
Histone exchange on the promoter region of *PMAIP1* by INO80 knockdown. (**A**) Quantitative PCR analysis of *PMAIP1* mRNA expression in INO80 knockdown cells. (**B**) Quantitative ChIP-qPCR analysis of *PMAIP1* promoter using an H2A.Z. antibody upon INO80 knockdown. (**C**) Quantitative ChIP-qPCR analysis of *PMAIP1* promoter using an H2A antibody upon INO80 knockdown.

Among the apoptosis-related genes, the expression of *PMAIP1* mRNA significantly increased by INO80 knockdown (Fig. 5A). *PMAIP1* is a member of pro-apoptotic subfamily of the BCL-2 protein family and is a known target of p53. *PMAIP1* is a tumor suppressor gene candidate in the pancreatic cancer cell line^15^ and induces apoptosis in lung cancer^16^. PMAIP1 induces apoptosis in several cell lines^17^.

To elucidate the role of INO80, we examined whether INO80 altered chromatin modifications in the promoter region of *PMAIP1*. We examined the role of INO80 in the promoter region because INO80 complex occupies the nucleosome free regions of the transcriptional start sites of over 90% in the budding yeast promoters^18^. Enrichment of INO80 at the promoter regions was correlated with transcriptional activity^19^. Chromatin immunoprecipitation (ChIP) was performed on HK-2 cells with *INO80* knockdown using the H2A.Z. antibody, because INO80 has been reported to remove the H2A.Z. variant and exchange it for the H2A variant^20,21^. When qPCR was performed on collected DNA samples using primers for the promoter regions of *PMAIP1*, INO80 knockdown significantly increased H2A.Z. in the promoter region of *PMAIP1* (Fig. 5B). In contrast, H2A expression significantly decreased in the promoter region of *PMAIP1* (Fig. 5C). We also confirmed that the H2A.Z. in the promoter region of *TP53* also significantly increased and H2A decreased when INO80 is knocked down (Fig. S6A). INO80 knockdown significantly increased H2A.Z. in the promoter region of *E2F1* while H2A has a tendency of decrease (*p* value = 0.07) (Fig. S6B). These results suggested that INO80 removed H2A.Z. and exchange it for H2A in the promoter region of apoptosis-related genes, resulting in the inhibition of apoptosis.

## Discussion

Here, we demonstrated the functional roles of INO80 in renal tubular cells. The novel findings of this study are as follows: (1) INO80 decreases under hypoxia and kidney injury; (2) INO80 protects renal tubular cells from apoptosis; (3) INO80 has histone-exchange activity that can replace nucleosomal H2A.Z./H2B with H2A/H2B in the promoter region of *PMAIP1*, resulting in increased apoptosis of tubular cells via the accumulation of H2A.Z. and the reduction of H2A levels in injured kidney cells.

Chromatin remodeling complexes are evolutionarily conserved from *Saccharomyces cerevisiae* to humans. Several chromatin remodeling complexes contain actin and actin-related protein as essential components for their function^22^. Chromatin remodeling complexes are transported to specific chromatin regions by histone variants, histone modifications, or interactions with transcription factors. Thereafter, the removal of nucleosomes, conversion of positioning, exchange of histone variants in nucleosomes, and changes in the histone modification state are performed in an ATP-dependent manner to change the chromatin structure at the nucleosome level. H2A.Z. is a H2A histone variant that is conserved from yeast to humans, suggesting that H2A.Z. has important biological roles^23^. Previous studies have shown that H2A.Z. contributes to epigenetic regulation in various species^24,25^. For example, H2A.Z. are located in the promoter regions of genes and are involved in chromosomal distribution and DNA damage repair. In our model, H2A.Z. accumulated and H2A was reduced when INO80 was knocked down in HK-2 cells in the promoter regions of *PMAIP1.* In *Arabidopsis*, the INO80 chromatin complex has been reported to promote thermomorphogenesis via H2A.Z. eviction and transcription^26^. In yeast, INO80 is recruited to specific promoters required for H2A.Z. exchange and it promotes hyphal development of *Candida albicans*^27^. Previous studies indicated that INO80 removes H2A.Z. and regulates its downstream target genes. In addition, INO80 was demonstrated to translocates along the DNA at the H2A-H2B interface of nucleosomes and persistently displace DNA from the surface of H2A-H2B^28^. INO80 has a dimeric exchange mechanism, H2A.Z. for H2A in an in vitro assay.

Several studies on INO80 and apoptosis have been conducted in oncology field. For example, in human colon cancer cells, INO80 knockdown increases apoptosis^29^ and in cervical cancer cells, INO80 knockdown does not influence apoptosis^30^. We hypothesized that INO80 may have different mechanisms depending on the cell type. In our experimental design, INO80 inhibited apoptosis of renal tubular cells during the progression of CKD. Although INO80 has been reported to be associated with renal function in a genome-wide analysis^9^, the mechanism by which INO80 works in the kidney and affects renal function has not been elucidated. To the best of our knowledge, this is the first study to show that INO80 inhibits tubular cell apoptosis by exchanging H2A.Z.

*PMAIP1*, a pro-apoptotic protein, is a member of the B-cell lymphoma 2 (Bcl-2) family and belongs to the Bcl-2 homology 3 (BH3) only protein subclass. Induction of *PMAIP1* occurs via both p53-dependent and p53-independent mechanisms. P53-independent *PMAIP1* regulators include hypoxic stimulation and the transcription factor E2F1. In addition to the p53 binding sites, the *PMAIP1* promoter contains a Hypoxia-Responsive Element (HRE) and an E2F1 binding site. *PMAIP1* was reported as a direct target of HIF1 and mediates hypoxic cell death in a p53-independent manner^31^. In our study, *PMAIP1* expression was found to be dynamically regulated by histone exchange via chromatin levels.

The current research has several limitations. Although we identified 35 INO80 down-stream target genes, the functions of the remaining down-stream target genes, except *PMAIP1* remain unknown. There is a possibility that more down-stream target candidates of INO80 were identified when we set different criteria in the analysis of RNA-seq. Further analysis of INO80 functions in chromatin remodeling will be needed.

## Conclusion

**Figure 6:**
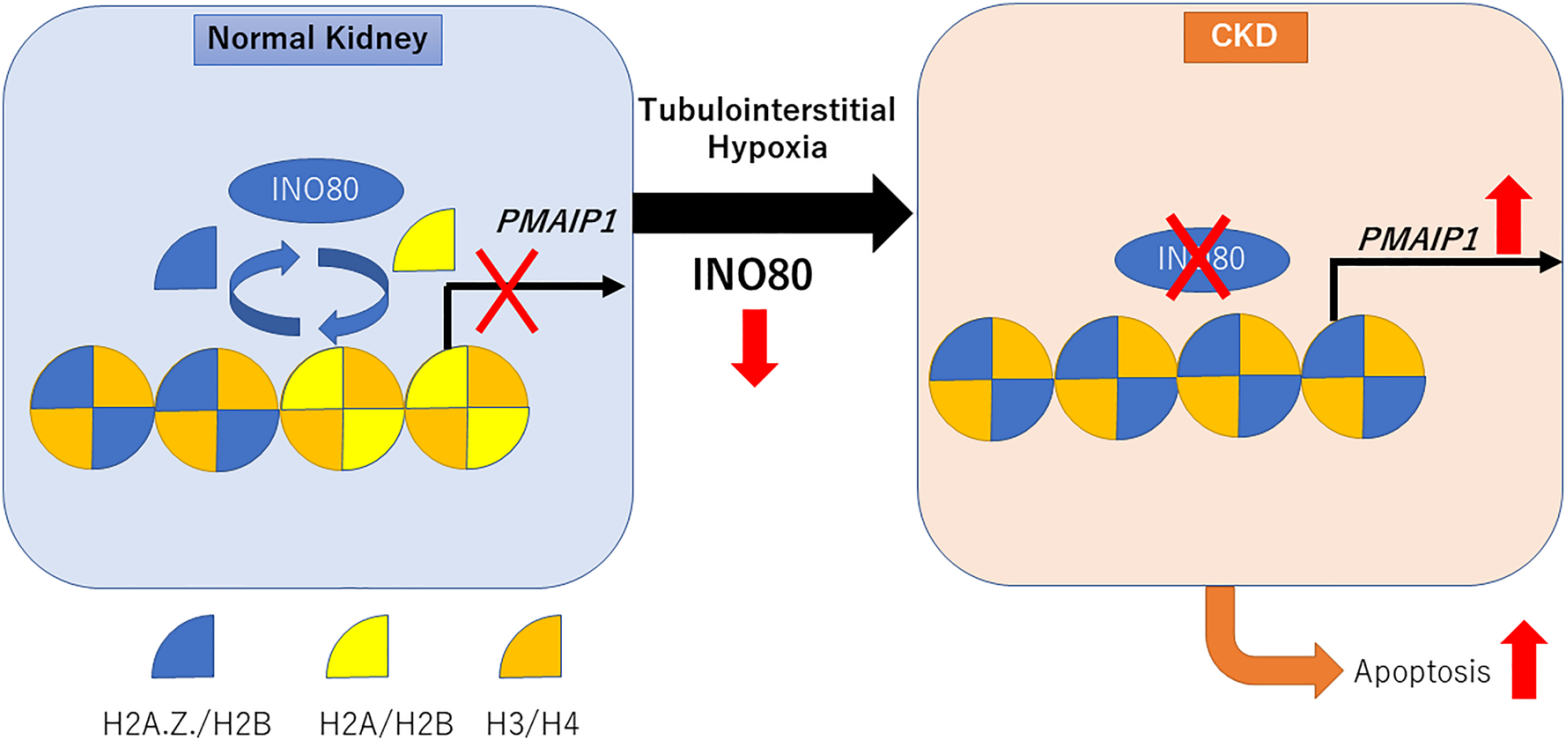
Schematic image of the role of INO80. In a normal kidney, INO80 removes H2A.Z. and places H2A on the promoter region of *PMAIP1*, suppressing PMAIP1 and inhibiting tubular cell apoptosis. During CKD progression, tubulointerstitial hypoxia decreases the expression of INO80, resulting in an increase in H2A.Z. and *PMAIP1*, leading to the promotion of apoptosis in tubular cells.

We found that INO80 levels decreased in patients with CKD. In normal kidney tissue, INO80 removes H2A.Z. and exchanges it for H2A on the promoter region of *PMAIP1*, suppressing *PMAIP1*, and inhibiting tubular cell apoptosis. However, during the progression of CKD, tubulointerstitial hypoxia decreases the expression of INO80, resulting in an increase in H2A.Z., which increases *PMAIP1*, leading to the promotion of tubular cell apoptosis (Fig. 6).

## Methods

### Cell culture and hypoxic environment

HK-2 (human kidney-2: ATCC CRL-2190) was purchased from American Type Culture Collection (ACTT). HK-2 cells were cultured in Dulbecco’s modified Eagle’s medium supplemented with F12 (Wako, Osaka, Japan) and 10% heat-inactivated fetal bovine serum (FBS). Cells were grown in a humidified atmosphere with 5% CO_2_ at 37 °C. Hypoxic conditions (1% O_2_ for 24 or 48 h for HK-2 cells) were created using a hypoxic cultivation incubator (ASTEC APM-30D, Fukuoka, Japan). The anoxic condition (0.1% O_2_) was created using an anoxia bag (Anaerocult® A Mini Gas-generating system, Merck Millipore, Japan or AnaeroPack and anaerobic cultivation sets, Mitsubishi Gas Chemical, Tokyo, Japan).

### Total RNA isolation and reverse transcription PCR

Total RNA was isolated using RNA iso Plus (Takara, Shiga, Japan), according to the manufacturers protocol. First-strand cDNA was synthesized using RT Master Mix (Takara, Shiga, Japan).

### Quantitative real-time PCR

cDNA was subjected to real-time quantitative PCR using THUNDERBIRD^®^ qPCR Mix (Toyobo Co. Ltd., Osaka, Japan) and a CFX96 Real Time System (Bio-Rad Laboratories Inc., Hercules, CA, USA), according to the manufacturer’s protocol. The results were analyzed using the ΔΔCt method and normalized with β-actin. The primer sequences are shown in Table 2.

**Table 2:** List of primers used in this study.

Target	Species		F/R	Sequence (5′ → 3′)
β-actin	Human	RT-qPCR	Forward	TCCCCCAACTTGAGATATATGAAG
			Reverse	AACTGGTCTCAAGTCAGTGTACAGG
VEGF	Human	RT-qPCR	Forward	CCCTGATGAGATCGAGTACATCTT
			Reverse	TCTGAGCAAGGCCCACAGGGA
INO80	Human	RT-qPCR	Forward	GGTCAAGTGGCAATACATGGT
			Reverse	CCGATTCCGACACTGGAACT
p53	Human	RT-qPCR	Forward	CAGCACATGACGGAGGTTGT
			Reverse	TCATCCAAATACTCCACACGC
E2F1	Human	RT-qPCR	Forward	CAAGAACCACATCCAGTGGC
			Reverse	GTCCTGACACGTCACGTAGG
PMAIP1	Human	RT-qPCR	Forward	CCTACTGTGAAGGGAGATGAC
			Reverse	CTGAAAAGCAAAACACCAAAA
β-actin	Rat	RT-qPCR	Forward	CTTTCTACATGAGCTGCGTG
			Reverse	TCATGAGGTAGTCTGTCAGG
INO80	Rat	RT-qPCR	Forward	TCAGAGAGACGGGACATGGT
			Reverse	GATTGATGCCCAGTCCTCCA
PMAIP1_ChIP	Human	ChIP-qPCR	Forward	TCCACAATGGGCGATATCGA
			Reverse	CCAGAGGCCCTGTGAGAAAG

### Knockdown of INO80 by siRNA

Cells were seeded at 50,000 cells per well in a 6-well plate and incubated for 24 h at 37 °C. Thereafter, they were transfected with Dharmacon ON-TARGET plus SMART pool siRNA (Horizon Discovery, Cambridge, UK) targeting INO80, and the negative control, Dharmacon ON-TARGET plus non-targeting pool (Horizon Discovery) using Lipofectamine™ RNAiMAX Reagent (Thermo Fisher Scientific) and Opti-MEM^®^ (Thermo Fisher Scientific). The cells were incubated at 37 °C and harvested after 48 h.

### Knockdown of HIF-1 by siRNA

The cells were seeded at 50,000 cells per well in a 6-well plate and incubated for 24 h at 37 °C. Thereafter, they were transfected with stealth RNAi™ siRNA targeting HIF-1 (Thermo Fisher Scientific), and the negative control, Stealth™ RNAi siRNA Negative Control Med GC (Thermo Fisher Scientific) by using Lipofectamine™ RNAiMAX Reagent (Thermo Fisher Scientific) and Opti-MEM^®^ (Thermo Fisher Scientific). The cells were incubated at 37 °C and harvested after 6 h. After being switched from Opti-MEM to normal medium, the cells were collected 24 h later.

### Animal experiments

#### Unilateral ureteral obstruction (UUO) rat

Six-week-old, male Wistar rats (Nippon Bio-Supp. Center, Tokyo, Japan) weighting 150–170 g were used in this study. After one week of acclimatization, the left ureter was ligated, and the rats were sacrificed 10 d after the operation. The anesthesia used for the operation was a combination of midazolam (4 mg/kg; Sandoz, Tokyo, Japan), butorphanol (1 mg/kg, Meiji Seika Parma Co., Ltd., Tokyo, Japan), and medetomidine (Kyoritsu Seiyaku, Tokyo, Japan) and was injected intraperitoneally. This study was approved by the Center for Disease Biology and Integrative Medicine. All the protocol was followed by “Regulations for the Conduct of Animal Experiments” and “Manual for the Conduct of Animal Experiments” in the University of Tokyo. All persons involved in this animal experiment attended the training course of animal experiment in the University of Tokyo (Rika Miura: 17,253, Imari Mimura: 17,250). Animal experiments was conducted in accordance with the protocols approved by the Animal Experiment Committee and the Ethics Committee of the University of Tokyo (approve number P18-060). All methods are reported in accordance with ARRIVE guidelines.

### Western blotting

Whole kidneys and HK-2 cells were lysed using RIPA buffer (50 mM tris–HCl (pH 8.0), 150 mM sodium chloride, 0.5% sodium deoxycholate, 0.1% sodium dodecyl sulfate and 1.0% Triton X-100) with a protease inhibitor mixture (cOmplete^®^ Mini, Sigma) on ice. Whole-kidney samples were homogenized using a homogenizer (PHYSCOTRON NS-310EIII, MICROTEC Co. Ltd.). Both the whole kidney and cell samples were centrifuged at 6000 rpm for 3 min at 4 °C. The supernatants were collected as the total protein content. Protein concentrations were measured using the DC™ Protein Assay Kit (Bio-Rad Laboratories Inc.). The extracted protein solutions were denatured with incubation in sample buffer (60 mM Tris (pH 6.8), 2% sodium dodecyl sulfate, 10% glycerol, 10 mM dithiothreitol and 0.01% bromophenol blue) for 5 min at 95 °C. The lysates were separated with 8% sodium dodecyl sulfate–polyacrylamide gel electrophoresis under reducing conditions. After electrophoresis, the protein samples were transferred onto polyvinylidene difluoride membranes (Millipore). Nonspecific protein binding was blocked with 5% skim milk in Tris-buffered saline (pH 7.4), containing 0.05% Tween 20 (TBST). The membranes were subjected to immunoblotting using polyclonal rabbit anti-INO80 (1/2000 dilution; Abcam ab118787, Cambridge, UK) and polyclonal rabbit anti-β-actin (1/2000 dilution; Bio-Rad Laboratories Inc.) overnight at 4 °C. The membranes were incubated with horseradish peroxidase-conjugated goat anti-rabbit IgG antibody (1/10,000 dilution; Bio-Rad Laboratories Inc.) for 30 min at room temperature. The bands were developed with Pierce™ ECL Plus Western Blotting Substrate (Thermo Fisher Scientific) and detected with Image Quant LAS 4000 (GE Healthcare, Tokyo, Japan). The bands were normalized by β-actin. Quantitatively, we analyzed the average of 20 fields of image where the fluorescence signal intensity was divided by the number of nuclei and averaged using Image J.

### Immunohistochemistry

The formalin-fixed and paraffin-embedded tissues were prepared with 3 μm thick sections. The sections were dewaxed with Histoclear^®^ (National Diagnostics) and rehydrated using a graded series of ethanol. After washing with phosphate buffered saline (PBS), tissue slides were subjected for antigen retrieval by boiling in 10 mM citrate buffer (pH 6.0) for 10 min in a microwave. Endogenous peroxidase activity was quenched with 3% H_2_O_2_ in PBS for 15 min at 24 °C. Nonspecific antibody binding was blocked via incubation with Protein Block Serum-Free (Dako, Agilent Technologies) for 15 min at 24 °C and subsequently incubated overnight at 4 °C with primary antibody, polyclonal rabbit anti-INO80 (1/400 dilution; LSBio LS-B11393) (1/400 dilution; Abcam ab118787). Thereafter, the sections were incubated with a biotinylated, affinity-purified anti-immunoglobulin secondary antibody (PK-6101 VECTASTAIN Elite ABC Rabbit IgG Kit; Vector Laboratories Inc., Burlingame, CA, USA), for 40 min at 24 °C. The color was developed using ImmPACT™ DAB (Vector Laboratories Inc.). Finally, the samples were dehydrated and mounted on coverslips. A BX51 microscope (OLYMPUS, Tokyo, Japan) was used for observation, and the slides were photographed using a camera DP20-5 (OLYMPUS).

Commercially available human kidney samples were obtained from ProteoGenex (CA, USA), and an outline of the samples is shown in Supplementary Table S1.

### Immunocytochemistry

Cells were seeded at 20,000 cells per dish in 35 mm diameter glass-based dishes and incubated at 37 °C for 24 h. Thereafter, they were fixed with methanol and acetone 1:1 for 30 min on ice. After washing with PBS, cells were incubated with 0.3% Tween 20 in PBS for 30 min at 24 °C. Non-specific antibody binding was blocked via incubation with 5% bovine serum albumin, diluted with PBS for 30 min, and Protein Block Serum-Free (Dako by agent Technologies) for 10 min at 24 °C. They were incubated for 1 h at 24 °C with the primary polyclonal rabbit anti-INO80 (1/400 dilution; Abcam ab118787). After washing with 0.1% Tween 20, the membranes were incubated with a secondary polyclonal swine anti-rabbit immunoglobulins/FITC (1/20 dilution; Dako Agent Technologies) for 1 h at 24 °C in dark. Finally, the cells were loaded with Hoechst stain (1/2000 dilution; bisbenzimide H33342 trihydrochloride, Sigma Aldrich, St. Louis, MO, USA) for 2.5 min for nuclear staining, followed by coverslip mounting. Fluorescence microscope BZ-X710 (KEYENCE, Osaka, Japan) was used for observation. For quantitative analysis, 20 fields of view were photographed with the 1000 × lens of a fluorescence microscope, and the fluorescence signal intensity in each nucleus was measured using ImageJ software^32^.

### Functional assay

#### Caspase 3/7 assay

We measured caspase 3/7 activity using the Caspase-Glo^®^ 3/7 Assay kit (Promega, Madison, WI, USA), according to the manufacturer’s protocol. This is a homogeneous, luminescent assay that measures caspase 3 and caspase 7 activities. Luminescence is proportional to caspase activity. First, HK-2 cells were transfected with siRNA and reseeded in 96-well plates at 100,000 cells per well in 100 μL of FBS-free medium. Cells were cultured under normoxic or hypoxic conditions for 48 h. Thereafter, we added the 100 μL of caspase-glo reagent, which is a mixture of caspase-glo substrate and caspase-glo buffer. Luminescence was measured after 30 min of incubation using luminometer (Multimode Plate Reader EnSpire^®^, PerkinElmer, Inc., MA, USA).

#### MTS assay

We evaluated cell viability using CellTiter^®^ 96 AQueous One Solution Cell Proliferation Assay (Promega), according to the manufacturer's protocol. The assays were performed by adding the MTS reagent directly to culture wells, and the quantity of formazan product, as measured by the absorbance at 490 nm, was directly proportional to the number of living cells in the culture. First, HK-2 cells were transfected with siRNA and reseeded in 96-well plates at 100,000 cells per well in 100 μL of FBS-free medium. Cells were cultured under normoxic or hypoxic conditions for 24 h. After adding 20 μL of MTS reagent and incubating at 37 °C for 1 h, absorbance at 490 nm was measured using a plate reader (Multimode Plate Reader EnSpire^®^, PerkinElmer, Inc).

### Chromatin immunoprecipitation (ChIP)

HK-2 cells in which INO80 was knocked down using siRNA, were seeded in 6-well plates**.** HK2 cells were cross-linked with 1% formaldehyde for 10 min. After neutralization using 0.2 M glycine, cells were collected, re-suspended in SDS lysis buffer (10 mM Tris–HCl, 150 mM NaCl, 1% SDS, 1 mM EDTA; pH 8.0, Protease inhibitor cocktail), and fragmented using a sonicator (SONIFIER^®^ CELL DISRUPTOR 350, Tokyo, Japan; 5 min, 60% duty, output control 7). The sonicated solution was diluted with ChIP dilution buffer (20 mM Tris–HCl (pH 8.0), 150 mM NaCl, 1 mM EDTA, 1% Triton X-100) up to 3 mL, which was used for immunoprecipitation (2.7 mL) and the remaining 300 μL as a non-immunoprecipitated chromatin (INPUT). Specific antibodies were bound and applied to the diluted sonicated solutions for immunoprecipitation. Antibodies against H3K4me3 (MABI0304, Nagano, Japan) were used in combination with magnetic beads (Dynabeads™M-280 sheep anti-Mouse IgG; 11201D, Invitrogen™, CA, USA). The prepared DNA was then processed for qPCR analysis. The primers used for ChIP-qPCR are listed in Table 2.

### RNA sequencing

mRNA was isolated as described above. RNA-seq libraries were prepared and sequenced using the HiSeq platform (Illumina, San Diego, CA) according to the manufacturer’s protocol. The reads per kilobase of exons per million mapped reads for each gene were calculated based on the length of the gene and read counts mapped to the gene. The sequences were aligned with human reference genome (UCSC hg38) using ELAND (Illumina)^33^. Details were described in our previous paper^34,35^. GENEWIZ (South Plainfield, NJ, USA) was used to analyze mRNA samples extracted from HK-2 cells with INO80 knockdown. RNA samples were prepared using the RNeasy Mini Kit (Qiagen, Hilden, Germany) according to the manufacturer's instructions.

### Statistical analyses

All measurements were expressed as the mean ± SEM. Data between groups were compared using a Student’s two-tailed *t* test. Statistical significance was set at *p* < 0.05. All analyses were performed using Microsoft Excel and GraphPad Prism 9.

### Supplementary Information

Supplementary Information.

## Data Availability

The RNA-seq data in this manuscript have been uploaded in Genome Expression Omnibus of NCBI. The GEO accession number is GSE224479. Any additional data supporting the findings of this study are available from the corresponding author on reasonable request.
