# Supplementary material for: Chromatin remodeling factor, INO80, inhibits PMAIP1 in renal tubular cells via exchange of histone variant H2A.Z. for H2A
Source: Sci Rep. 2023 Aug 14;13:13235. doi: 10.1038/s41598-023-40540-8 (PMC10425331; doi:10.1038/s41598-023-40540-8)
Supplement: Supplementary file 1 — Supplementary Information. [file 41598_2023_40540_MOESM1_ESM.pdf]

Supplementary Figures and Tables.

Chromatin remodeling factor, INO80, inhibits *PMAIP1* in renal tubular cells via exchange of histone variant H2A.Z for H2A.

Authors: Rika Miura, Imari Mimura, Hanako Saigusa, Tomotaka Yamazaki, Fumiaki Tanemoto, Yu Kurata, Dai Sato, Tetsuhiro Tanaka, Masaomi Nangaku

Supplementary Figures and Legends.

Fig S1. VEGF mRNA expression under 24h hypoxia and 24 h anoxia.

Quantitative PCR analysis of *VEGF* mRNA expression under normoxia vs. hypoxia and anoxia for 24 h in HK-2 cells. Data are shown as the mean and the standard deviation from triplicates.

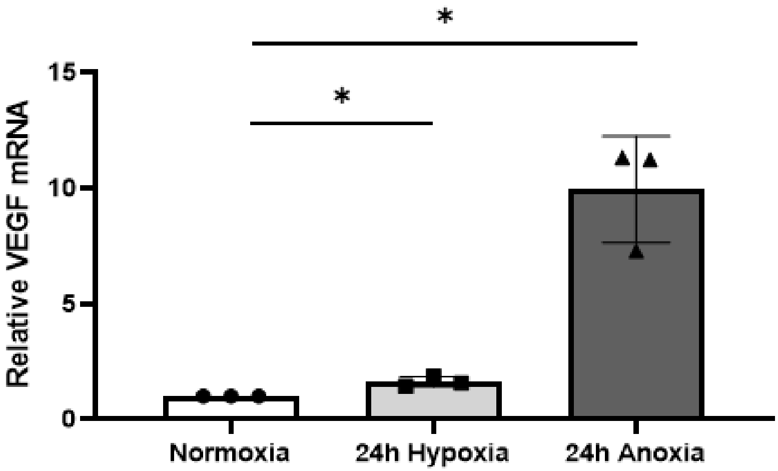

Fig S2. **Multiple exposures for full membrane of western blotting using INO80 and β-Actin antibodies.**

Western blot of INO80 extracted from HK-2 cells under normoxic condition, 1% hypoxia and anoxia for 24 h. The membrane was cut because we needed longer exposure time to detect INO80 than to detect β-Actin. The right lanes were protein samples in which siRNAs of control and INO80 were used to identify the INO80 band. WB for INO80 is exposed for 8 seconds, 1min, and 4 minutes. Figure 1B in the revised manuscript is 4 minutes exposure.

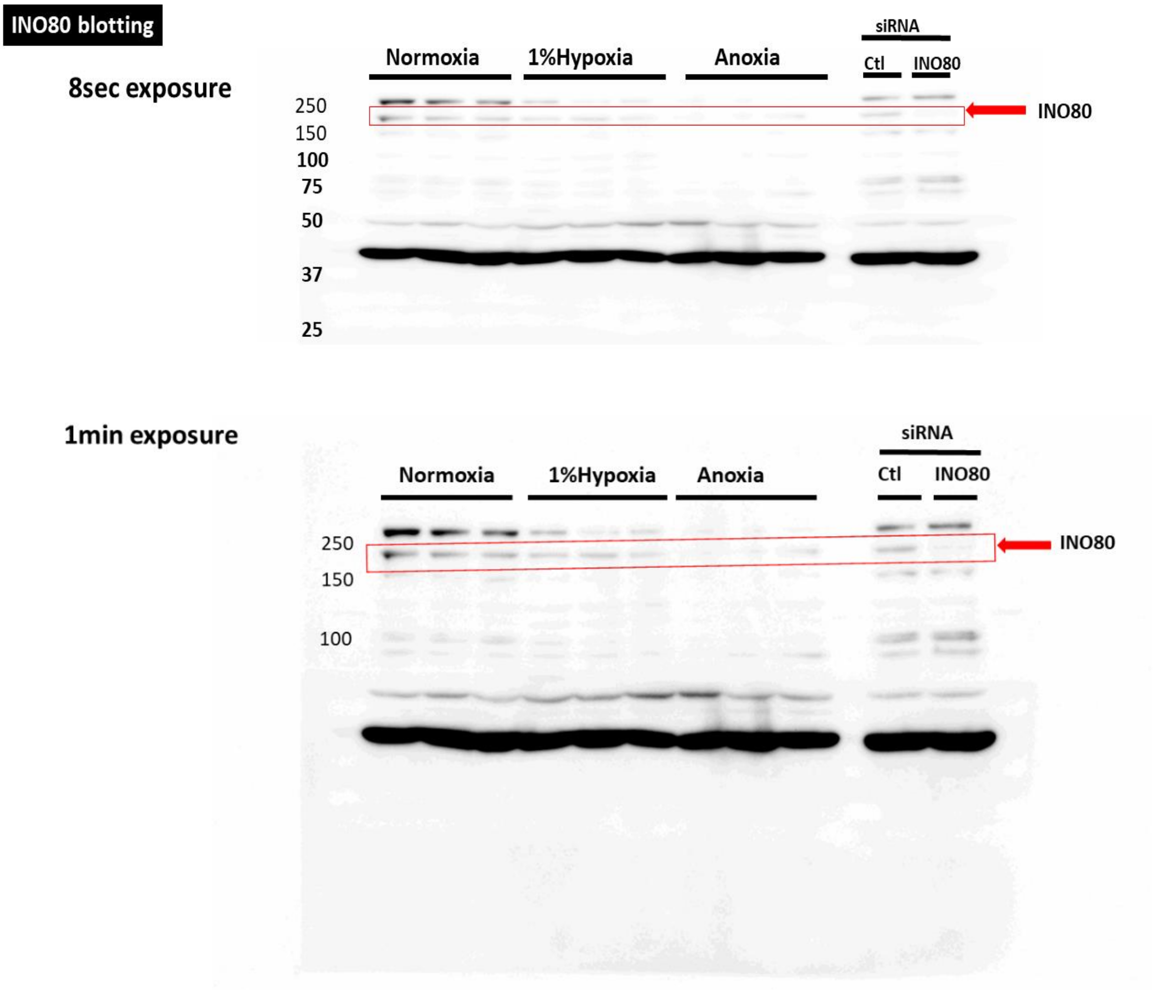

4min exposure

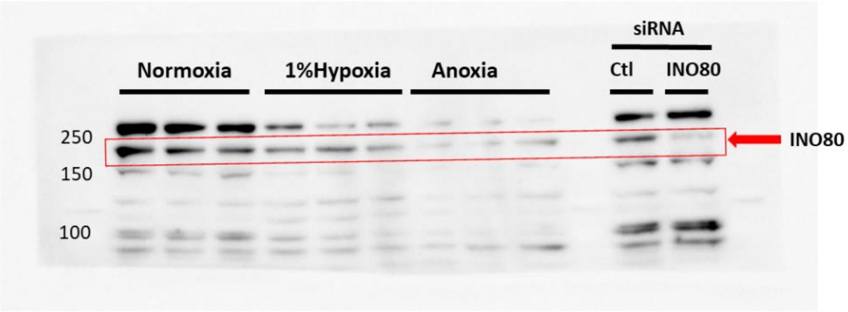

βACTB blotting

8sec exposure

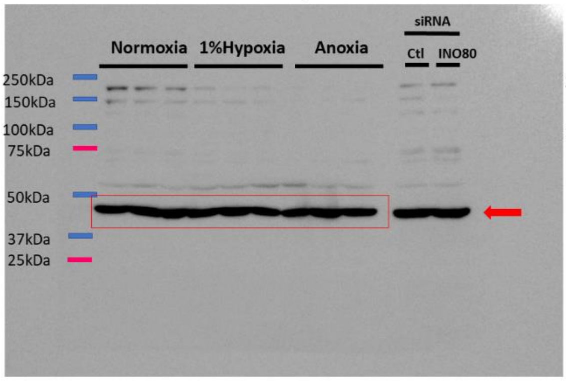

1min exposure

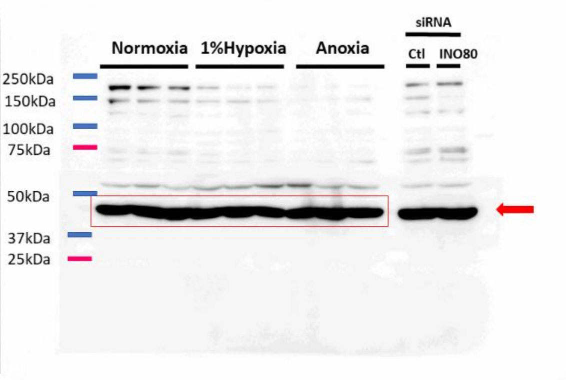

Fig S3. INO80 is down-regulated in impaired kidneys.

(A) Quantitative PCR analysis of *INO80* mRNA expression in the UUO rat kidneys. Data are shown as the mean and the standard deviation from n=3.

(B) Representative images of Masson trichrome staining of 5/6 Nx rat kidneys. Scale = 100 μm, x400.

(C) Representative immunohistochemistry images of INO80 staining of 5/6 Nx rat kidneys. Scale = 100 μm, x400.

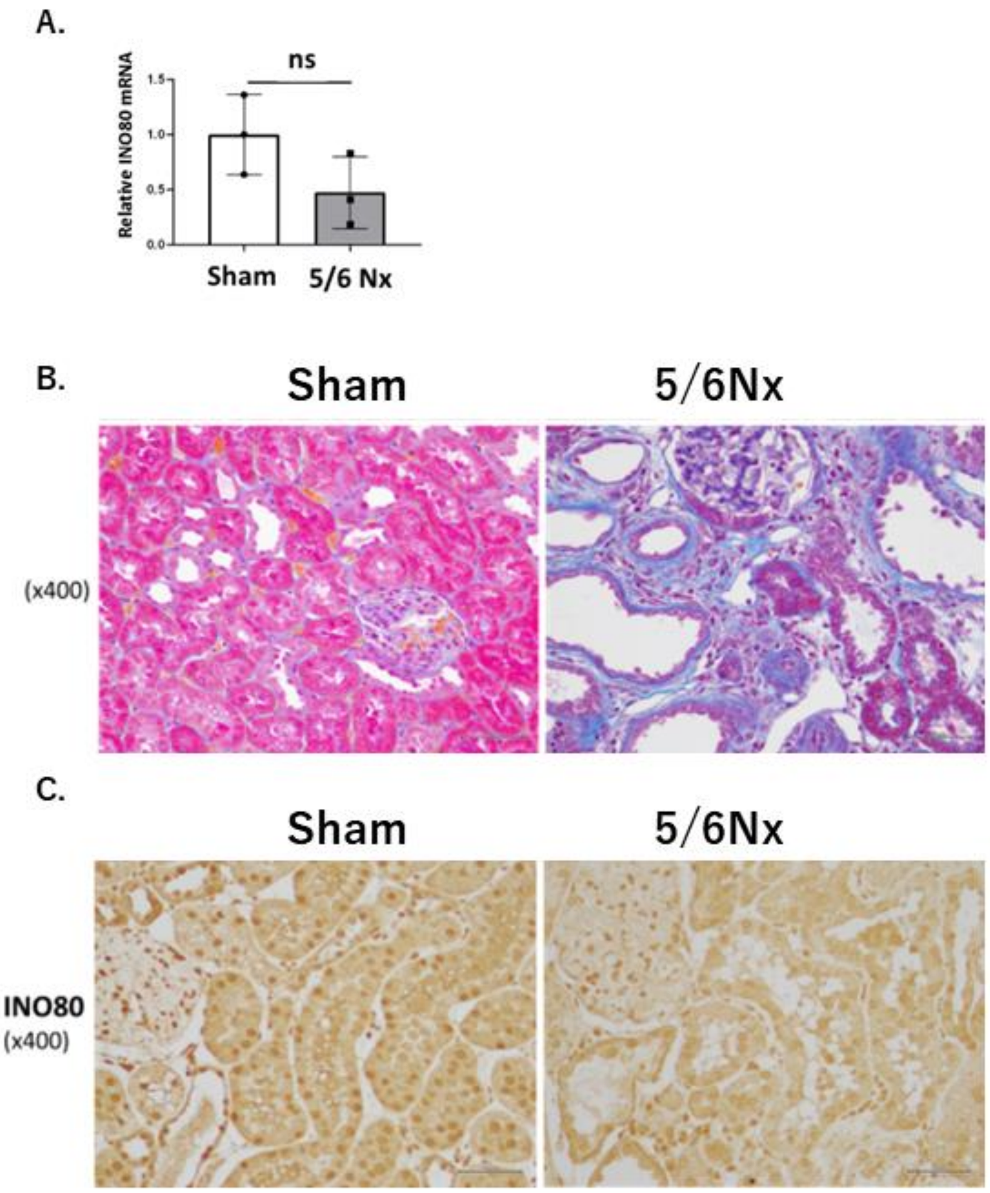

Fig S4. INO80 is down-regulated in impaired kidneys.

Immunohistochemistry image of INO80 in human kidney samples. See Table S1 for these samples background. Scale = 50 μm, x200.

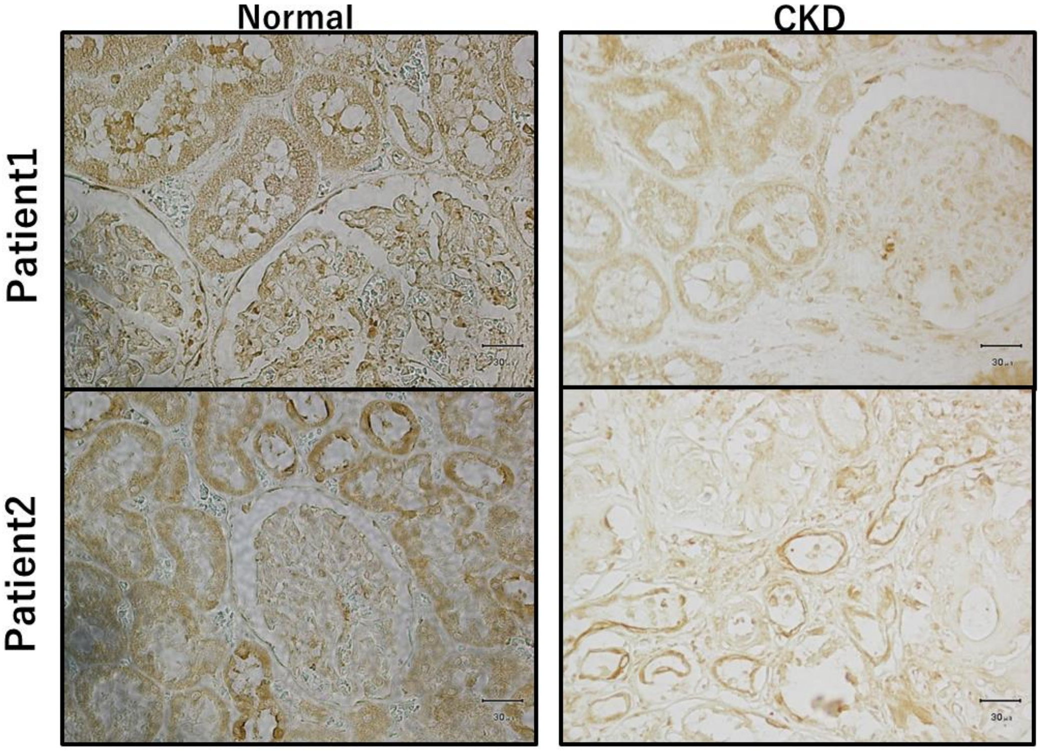

Fig S5. Multiple exposures of full membranes of western blotting of β-actin extracted from HK-2 cells under normoxic condition, 1% hypoxia and anoxia for 24 h.

WB for INO80 is exposed for 15 seconds, 1min, and 4 minutes. WB for β-ACTB are exposed for 15sec and 1min. Figure 3B in the revised manuscript is 4 minutes exposure for INO80 and 15 seconds for β-ACTB.

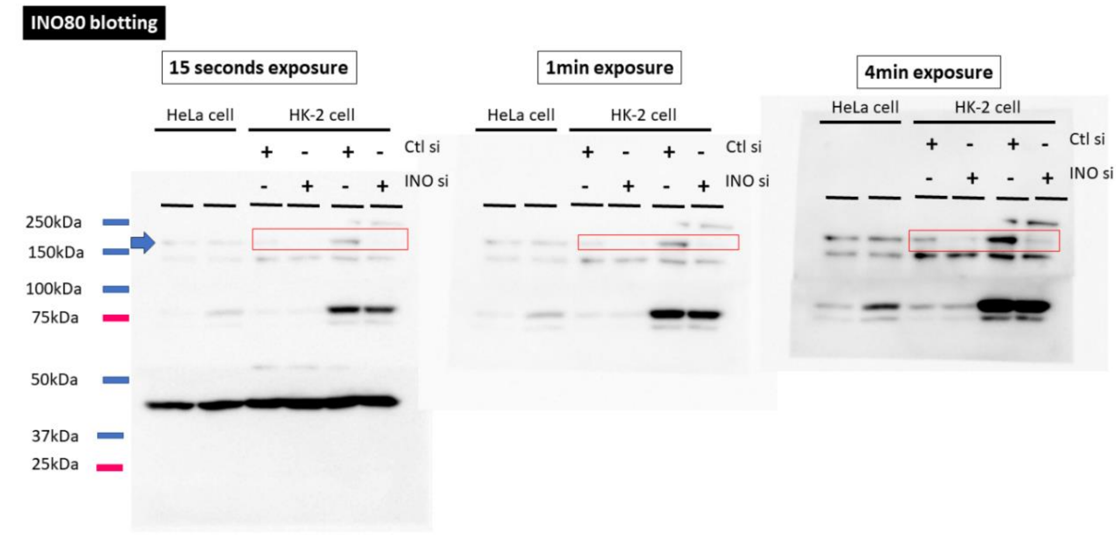

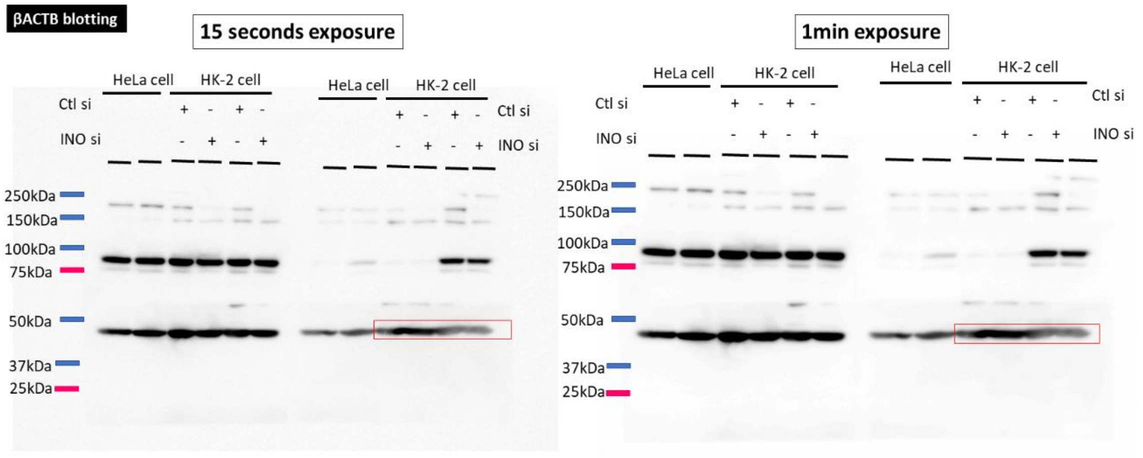

Fig S6. Histone exchange on the promoter region of TP53 and E2F1 by INO80 knockdown.

(A) Quantitative ChIP-qPCR analysis of TP53 promoter using an H2A.Z. antibody and H2A antibody upon INO80 knockdown. Representative data of three independent experiments. *p<0.05

(B) Quantitative ChIP-qPCR analysis of E2F1 promoter using an H2A.Z. antibody and an H2A antibody upon INO80 knockdown. Representative data of three independent experiments. *p<0.05

A.

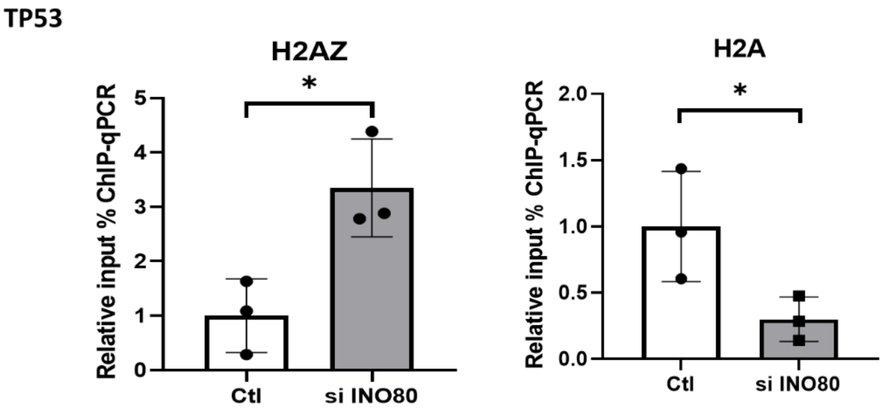

B.

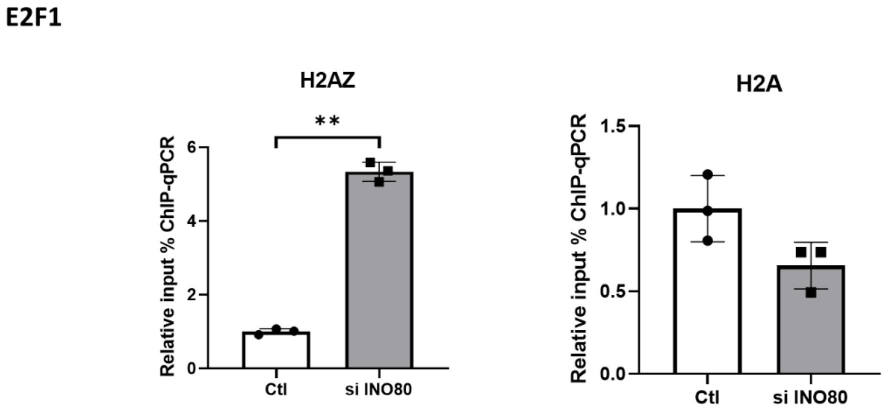

Supplementary Table S1. Outline of human samples

	Diagnosis	ID	sex	age	Ethnic background	Matrix	Date of autopsy	BMI	Cause of Death
Patient1	Normal	051744A	M	32	Caucasian	kidney	07/16/2015	41.5	neck phlegmon, PE
Patient2	Normal	051763A	M	35	Caucasian	kidney	08/14/2015	24.7	perforation of peptic ulcer, peritonitis
Patient1	CKD	051917A	M	84	Caucasian	kidney	06/09/2016	24.5	discirculatory encephalopathy, pneumonia
Patient2	CKD	051950A	M	71	Caucasian	kidney	11/16/2016	27.8	CKD

Supplementary Table S2. Primers for ChIP-qPCR.

TP53_ChIP	Human	ChIP-qPCR	Forward	AAGGGCAAGTAATCCGCCTG
			Reverse	TCCACCAATTCTGCCCTCAC
E2F1_ChIP	Human	ChIP-qPCR	Forward	AAGAGCAGCAGGTCAGGGTC
			Reverse	AGATCGTCATCATCTCCGCC
